# Label-Free Detection of Neuronal Differentiation in Cell Populations Using High-Throughput Live-Cell Imaging of PC12 Cells

**DOI:** 10.1371/journal.pone.0056690

**Published:** 2013-02-22

**Authors:** Sebastian Weber, María L. Fernández-Cachón, Juliana M. Nascimento, Steffen Knauer, Barbara Offermann, Robert F. Murphy, Melanie Boerries, Hauke Busch

**Affiliations:** 1 Freiburg Institute for Advanced Studies (FRIAS), Albert-Ludwigs-University Freiburg, Freiburg, Germany; 2 Center for Biological Systems Analysis, Albert-Ludwigs-University Freiburg, Freiburg, Germany; 3 Lane Center for Computational Biology and Department of Biological Sciences, Carnegie Mellon University, Pittsburgh, Pennsylvania, United States of America; INSERM U894, France

## Abstract

Detection of neuronal cell differentiation is essential to study cell fate decisions under various stimuli and/or environmental conditions. Many tools exist that quantify differentiation by neurite length measurements of single cells. However, quantification of differentiation in whole cell populations remains elusive so far. Because such populations can consist of both proliferating and differentiating cells, the task to assess the overall differentiation status is not trivial and requires a high-throughput, fully automated approach to analyze sufficient data for a statistically significant discrimination to determine cell differentiation. We address the problem of detecting differentiation in a mixed population of proliferating and differentiating cells over time by supervised classification. Using nerve growth factor induced differentiation of PC12 cells, we monitor the changes in cell morphology over 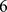 days by phase-contrast live-cell imaging. For general applicability, the classification procedure starts out with many features to identify those that maximize discrimination of differentiated and undifferentiated cells and to eliminate features sensitive to systematic measurement artifacts. The resulting image analysis determines the optimal post treatment day for training and achieves a near perfect classification of differentiation, which we confirmed in technically and biologically independent as well as differently designed experiments. Our approach allows to monitor neuronal cell populations repeatedly over days without any interference. It requires only an initial calibration and training step and is thereafter capable to discriminate further experiments. In conclusion, this enables long-term, large-scale studies of cell populations with minimized costs and efforts for detecting effects of external manipulation of neuronal cell differentiation.

## Introduction

Neuronal differentiation and morphogenesis have been a subject of intense research during the last decades [1]. A central question is the elucidation of the intricate orchestration of signaling on the proteome and transcriptome levels that controls the decision between proliferation and differentiation of neuronal progenitor cells [2]–[4]. Much research in the field of neuronal cell research has focused on characterizing neurite growth of single cells by measuring average neurite length or the number of branching points [5], [6]. However, this leaves out the important question, under which treatment conditions differentiation of the whole cell population occurs. This is addressed in the following by means of an automated high-throughput data-driven analysis of live-cell imaging.

As a model system we use the neuroendocrine PC12 cell line. This is a popular substitute to study the processes of neuronal differentiation [7], since study on primary neuron cells is hindered due to the low yield of primary neurons from animal models and the difficulties of primary neuron cell culture. The popularity of PC12 cells originates from their ease of handling, ability to expand indefinitely, and relative high transfection capability [8]. Upon stimulation with nerve growth factor (NGF), PC12 cells change their morphology by flattening and growing neurites, resembling the phenotype of sympathetic ganglion neurons.

Despite the progress in deciphering the early molecular events that decide between proliferation or differentiation within PC12 cells [2], [4], [9], a thorough classification of the differentiation status of the whole cell population based on cell morphology still remains challenging. For more than 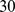 years, the state of the art has been the manual or semi-automated measurement of neurite formation from photomicrographs [10]. Neurite measurements are time and labor intensive, as they require tuning and adaptation to the respective experiment as well as frequent interventions in the semi-automated case. Moreover, this approach is error prone, as under NGF stimulation PC12 cells tend to simultaneously differentiate and proliferate by growing in clumps. This can make it hard to manually detect enough single cells suitable for neurite measurements [11]. Nonetheless, these methods are still utilized extensively in many research laboratories due to the relatively low costs and ease of implementation [12]–[15].

Automated image analysis using fluorescently labeled cells to visualize neurite outgrowth/length has gained popularity in recent years [16]–[18]. The differentiation status is derived from the relation of cell body diameter to neurite length, which, however, requires both single, individual cells as well as a sufficient fluorescent signal [19]–[21]. While the advantage of high signal-to-noise ratio in fluorescently labeled cells is obvious, there are disadvantages associated with immunofluorescence as well. In general, immunofluorescence is performed either on fixed or live cells. The former is a terminal analysis, disallowing temporal follow-up studies, while the latter requires extra steps of transfection and involves possible risks of photo-toxicity. This can influence cell homeostasis and constitutes an extra source of error due to heterogeneous transfection rates, which can be a problem for primary neurons in particular.

Work has also been done on automated analysis of phase contrast images to measure neuronal morphology changes without the need for fluorescent markers [5], [6], [22]. These approaches focused on explicit detection of developmental changes of single neurites that requires continuous monitoring of individual cell plates. Thus, parallel monitoring of differently treated cell populations with the same microscopic device becomes more complicated. Moreover, these approaches require low cell densities or even single cells as well as manual adjustment of many parameters, i.e. even per cell plate and/or cell location. Hence, scalability of these approaches to the cell population level and/or compound or parameter screening is limited.

To cope with these biological and technical difficulties in detecting differentiation of neuronal cell populations, we present an adaptive, automated machine learning approach based on supervised classification using an initially broad image feature set. In fact, similar supervised machine learning methods have been developed to determine cell viability from dark field microscopy images [23]. Here we show that our method is capable of detecting the differentiation status of live-cell populations irrespective of cell density and without the need for any cell interference such as fluorescent markers or staining. The method requires initialization with only 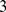 steps: (i) specification of three cell morphology parameters, (ii) measurement of a calibration data set to capture the characteristics of experimental factors like the microscope, and (iii) a measurement of training images of differentiated and undifferentiated cells. As a source of training data, we used unstimulated (CTL) and NGF-stimulated cells and monitored both over a period of six days after first stimulation. A large initial image feature set is used for robustness and to avoid bias under varying experimental conditions. The set of image features was first reduced by a feature selection step to achieve maximal classification performance for specific training images. We evaluated our final classifier on hold-out and biologically independent data sets. Since NGF simultaneously promotes differentiation, cell survival, and proliferation [24], we demonstrate that our system can measure differentiation independently of proliferation over time. We therefore validated our approach under enhanced or reduced proliferation conditions, using epidermal growth factor (EGF) and mitomycin treatment, respectively. Moreover, we ensured that the classifier approach is applicable to both high and low cell densities, which are typically encountered when studying primary neuron populations. We compared our approach to the prior (manual counting) state of the art technique, quantifying neurite lengths, and found equivalent results. In summary, we have developed a classifier capable of detecting differentiation within a mixed live-cell population of possibly densely growing differentiating and proliferating neurons using phase-contrast images. Automated feature selection provides maximal adaptability for different cell cultures and experimental conditions with the goal that our procedure can be applied in a broad spectrum of neuronal research.

## Materials and Methods

### Cell Culture and Stimulation

PC12 cells (ATTC, Middlesex, UK) were cultured in RPMI 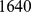 medium containing 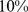 horse serum (HS), 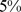 fetal calf serum (FCS), 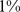 L-Glutamine and Penicillin/Streptomycin at 37°C in 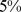 CO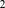. Cells were seeded on collagen-coated 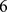-well plates (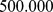 cells per well) for 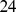 h before stimulation. PC12 cells were treated with 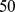 ng/ml rat Nerve Growth Factor-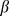 (NGF; Promega, Madison, WI, USA) to induce differentiation. Proliferation was stimulated via treatment with 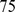 ng/ml human Epidermal Growth Factor (EGF; R&D Systems, Wiesbaden, Germany). The individual components were added every 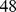 h during six days. To reduce cell proliferation, 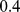 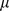
g/ml mitomycin c (Carl Roth, Karlsruhe, Germany) was added once for one hour and washed out before first stimulation. The mitomycin c concentration was chosen to ensure maximal cell viability at lowest proliferation rate, as shown by the low apoptosis even after 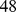 h of treatment (cf. Section ‘Quantification of apoptosis’ and Figure S1). Each condition (CTL 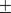 mitomycin, NGF 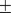 mitomycin and EGF 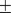 mitomycin) has been monitored in technologically independent duplicates. In addition, a second biologically independent experiment (cells originated from a different passage) with the treatments CTL, NGF and EGF has been carried out for validation purposes. To assess the effect of cell density and to test our classifier in a third independent experimental setup, we applied the NGF treatment to PC12 cells at a greatly reduced density of 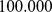 cells per well.

### Microscopic Imaging

Live phase-contrast images from PC12 cells under different conditions were acquired using a Nikon Eclipse Ti Inverted Microscope (Nikon Germany, Düsseldorf, Germany) equipped with a Perfect Focus System (PFS) and a cooled Digital Sight Camera (DS-QiMc; Nikon Germany, Düsseldorf, Germany). The recorded images were saved as 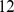 bit gray-scale with 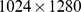 pixels at a 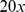 magnification and a resolution of 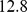 pixels per 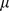
m. The images were acquired at defined positions on the motorized microscope table by a line-wise scanning of each well around its center, taking approximately five minutes per well. Before and after imaging one well the cells were placed back in the cell incubator. The primary data set was created by taking 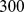 images, once every 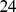 h for six days from two wells each under the six conditions. The second, biologically independent experiment was carried out on two wells each for conditions CTL, EGF and NGF (without mitomycin). Imaging was carried out with identical instrument parameters for the same time period of six days and the same 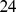 h interval. Per well and day 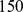 images were recorded for the secondary data set, unless stated otherwise. The third independent experiment was imaged with low cell density under that same conditions, yet for NGF and CTL alone and with 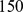 images per well. For calibration purposes we recorded a fourth data set of two collagen-coated wells without cells (containing just growth medium) over three days with 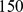 images per measurement.

### Immunostaining

Immunofluorescence labeling of PC12 cells was carried out as previously described [25]. Cells were cultured for five days in 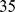 mm 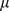-imaging dishes (ibidi; Munich, Germany). Cells were fixed with 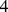
% paraformaldehyde on days 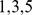 and subsequently permeabilized with 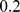
% TritonX in PBS for five minutes. Cells were labeled with a monoclonal anti-Tubulin antibody (diluted 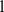: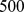; Sigma-Aldrich, St. Louis, MO, USA) for one hour. The secondary antibody Alexa Fluor 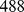-conjugated donkey anti-mouse immunoglobulin (diluted 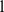: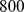; Invitrogen, Darmstadt, Germany) was incubated together with the nuclear marker DAPI (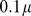
g/ml, Sigma-Aldrich, St. Louis, Mo, USA) for one hour. The resulting images were captured with a Nikon Eclipse Ti Inverted Microscope (Nikon Germany, Düsseldorf, Germany) equipped with a cooled Digital Sight Camera (DS-QiMc; Nikon Germany, Düsseldorf, Germany) using a 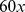 oil objective lens. Digitized images were processed by NLS software (Nikon Germany, Düsseldorf, Germany) and Adobe Illustrator (Adobe Systems).

### Quantification of Apoptosis

PC12 cells were treated with 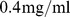 mitomycin c as described in the previous section or with 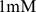 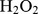 as a positive control. Apoptotic cell death was examined after 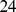 and 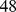 h according to the method of Nicoletti [26] using a Cyan ADP Flow Cytometer (Beckman Coulter; Miami, FL, USA). Three independent experiments were performed, where 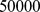 cells were counted and the results were presented as % of specific DNA fragmentation using the formula: (percentage of experimental apoptosis - percentage of spontaneous apoptosis)/(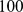 - percentage of spontaneous apoptosis) 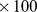.

### Quantification of Cell Differentiation by Human Counting

PC12 cell differentiation was determined on days 1 and 3 by manual measurements of the longest neurite length per cell, which was set in relation to the average cell diameter. Images were measured for the conditions NGF without and with mitomycin. On day 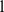 in total 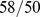 images with an overall of 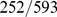 cells for without/with NGF were recorded and on day 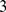 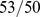 images with 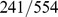 cells were counted respectively. A cell is defined to be differentiated if its longest neurite in relation to the average cell diameter is larger than 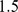.

### Image Features

We first filtered all images for outliers. A range filter counted all pixels, for which the difference in the maximal and minimal pixel intensity in a neighborhood of a 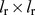 square per pixel was larger than a threshold 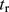. We required the number of counted pixels to be larger/smaller than the area corresponding to 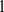 cell/the whole image area minus 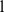 cell, where we set the cell area to 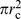 (cf. Table 1). This avoided empty or underexposed images, and completely filled images due to overexposure. In addition we required that at least one segment was detected by the ROI method below. We set the threshold 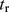 using the width of the 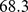
% quantile of the pixel intensity distribution (image feature 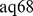), which we calculated for each image of the calibration data set. The distribution of this value differed between wells and days. We determined the mean value of the image feature 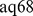 over all images per well and used the more conservative larger value of the two wells for the final threshold value 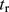. These rules identified a total of 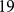 images as outliers, of which 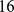 were found in one of the wells with NGF without mitomycin treatment. We verified these manually, and observed that these images were either recorded with very low contrast and out of focus or showed predominantly background. In order to assure proper stratification of the cross-validation scheme used later, we selected the other well to train the system.

**Table 1:** Parameters.

Constant	Value		Definition
Parameters depending on cell morphology			
*r_s_*	0.5	*µ*m	Radius of the disk used for the morphological fill in. Set to the length scale of a neurite; rounded to a radius of 6.5 pixel corresponding to a diameter of 13 pixel.
*r_c_*	2.5	*µ*m	Approximate lower bound of the cell radius, rounded to 32 pixel.
*t_a_*	50	pixel	Minimum number of pixels per segment (set to approximately one half of the area of the morphological fill in element, i.e. 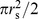 ).
Parameters set automatically by calibration data set			
*t_r_*	112	a.u.	Threshold above which the local range difference of a pixel is counted for the outlier detection in a 12bit image.
*t_e_*	0.0833	a.u.	Threshold for the strong edge detection of the Canny edge detector.
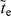	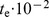	a.u.	Threshold for the weak edge detection of the Canny edge detector.
Parameters used in default setting of MATLAB			
*l*_m_	3	pixel	Side length of the square used as local neighborhood for the median filtering.
*l*_r_	3	pixel	Side length of the square used as local neighborhood for the range filtering.

Parameters used for image feature extraction.

Next, each image was filtered by a median filter to reduce noise (using a local neighborhood of a 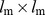 square). Then we identified regions of interest (ROI) containing cells using an edge detection and the image was thresholded by 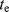. Afterwards, a morphological closure was applied with a structuring element of a disk having a radius of 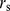 pixels, which was set to a value comparable to the size of a neurite. Finally, small artifacts of at most 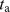 pixels were removed from the image. The obtained ROIs were then segmented into disconnected regions. To determine an optimal edge detection algorithm and an appropriate threshold 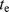 for the edge detection, we use the calibration data set described above. We evaluated the edge detection algorithms Canny, Prewitt, Sobel, Roberts and a Laplacian of Gaussians method. As selection criteria we required the edge detection to be robust against experimental factors, such as between- and intra-well variability. We determined the threshold 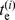 for each image 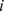 with the described method, which marks a fraction of 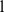
% of the image as ROI. The Canny algorithm required a second threshold 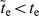 to detect weak edges. Because of this we considered the Canny edge detector in four variants. We set the second threshold 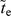 to 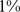, 5%, 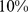 and 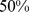 of the larger threshold 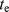 and evaluated the influence of the well and measurement day by a two-way ANOVA. This revealed a weak interaction of these factors and a significant difference in the mean threshold depending on the well and day. Evaluating the pairwise average differences between wells and different days, scaled by their standard deviations, showed that the difference between wells was larger than the differences per well over time (intra-well), i.e. each well was more homogeneous in time than it was to its paired well at a given time. Moreover, this comparison showed that the Canny edge detector with the second threshold 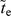 set to 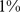 of 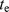 had the least difference across time. Hence, we selected this Canny edge detector and set the threshold 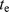 to the mean over all thresholds 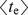 per well and selected the larger of the two values, since this will suppress background noise more robustly.

In summary we required eight constants to extract the image features, which are listed in Table 1. From these eight parameters three were determined by the size of a typical cell and an average neurite, three were calculated automatically from the calibration data set, and the remaining two are standard parameters used for image filtering. Finally, a set of 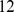 global image features, 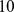 ROI-based features, and five per segment ROI image features were calculated (see Table 2). The latter features were summarized per image by taking the mean and standard deviation values over all segments in a given image. As many of the image features constitute count variables with a dependence of the variance on the mean value, we applied variance stabilizing transformations as suggested by Anscombe [27]. The image features 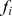 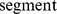, 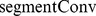, and 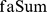 were limited by the total number of pixels 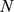 and were hence transformed by the 
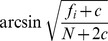
 transform suitable for Binomial variables. The parameter 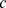 was set to 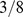 as recommended by Anscombe. All other variables related to areas and pixel intensities were assumed to constitute Poisson distributed count data and were transformed by the square root function. Entropy image features were 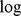 transformed.

**Table 2:** Feature Definition.

Feature	Definition
**Global**	
intensity	Average of the absolute deviation of each pixel intensity from the mean pixel intensity.
mean	Average pixel intensity.
med	Median pixel intensity.
std	Standard deviation of the pixel intensity.
mad	Median absolute standard deviation of the pixel intensity distribution.
Etot	Total entropy.
Emean	Average of the local entropy.
Emed	Median of the local entropy.
Estd	Standard deviation of the local entropy.
aiqr	Inter quartile range of the pixel intensity distribution.
aq68	Width of the central 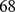 % quantile of the pixel intensity distribution.
aq95	Width of the central 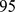 % quantile of the pixel intensity distribution.
**ROI dependent**	
segment	Area of the pixels determined to be ROI.
segmentConv	Area of the pixels after applying a convex hull covering to each segmented region.
skeleton	Total length of skeletonized ROI.
skeletonBranch	Number of branching points of skeleton.
skeletonEnd	Number of endpoints of skeleton.
endpoints	Endpoints of ROI image.
Euler	Euler number of image.
caSum	Total convex hull covering area summed over all segments (can become larger than 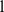 due to overlapping).
faSum	Total area of pixels within all segments after filled in holes.
numObj	Number of identified segments.
**Per segment ROI dependent, summarized by mean and standard deviation**	
solidity	Ratio of the filled area (with holes closed) and the convex hull area.
caRel	Convex hull covering area of the segment in percent of the segment size.
faRel	Area of pixels in the segment after filling in holes in percent of the segment size.
ecc	Eccentricity of a fitted ellipsoid covering the segment.
ext	Extent of the filled area of the segment.

Initial feature set considered for image analysis. The per segment calculated image features are summarized for each image by their mean and standard deviation. All image features that measure an area are recorded in percent of the total number of pixels. Pixel intensity related image features are given in percent of the maximal pixel intensity. For applied transformations see Material and Methods.

A well known technical artifact in phase-contrast microscopy is the spatial dependence of illumination intensity of culture well micrographs [17], which changes radially from the center to the edge. Together with a drift and readjustment errors of the microscope settings during the long-term observation over several days, this lead to an unique spatiotemporal correlation between the images in each measurement. This spatial correlation pattern translated by the line-wise scanning into an auto-correlated sequence of the recorded images. In fact, the mean image intensity varied periodically between 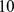
% and 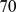
% of the maximal intensity. We restricted our analysis to the image features that are least affected by these auto-correlations. For this, we test for an AR(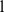) type autocorrelation with the Breusch–Godfrey test [28] in each image feature under the observed conditions. The 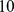 image features having the least auto-correlations were selected for downstream analysis.

### Supervised Image Classification

Clear examples of the undifferentiated and differentiated morphologies are needed to train the classifier. Since the rate and extent of differentiation may vary from experiment to experiment, we automated the choice of which day after stimulation to use as examples of the differentiated phenotype for training (and used the CTL cells from the same day as examples of the undifferentiated phenotype). To minimize the need for parameter choices, we applied the parameter-free Fisher’s linear discriminant analysis (FDA) and set up a nested and stratified cross-validation procedure to fix the remaining free parameters, the training day and the optimal set of features. We split the primary data set as follows: (i) A training set consisting of 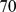
% of the images from one well of each condition, (ii) a hold-out/verification set, consisting of the remaining 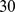
% of the images from that well, and (iii) all remaining images from the other well for each condition (to use as an independent set for testing of the final system). The latter data set constituted a technical replicate for verification. Finally, we used the secondary, biologically independent as well as the experimentally distinct data set as a further verification of the final approach.

For the nested cross-validation, we first split the training data set into 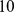 equally large folds for each stimulus condition. In the first step of the cross-validation, a feature selection out of the 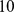 pre-filtered features that were robust against spatial intensity variability was performed. We selected each possible selection of eight out of nine folds for training and used the remaining fold for testing our performance criteria. The feature selection was implemented by a step-wise forward and backward search algorithm. Starting from an empty set, each feature not in the set was subsequently added and each feature in the set was subsequently removed. For each set the performance was determined by an 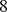-fold cross-validation of the correctness rate. The search algorithm stopped once the correctness rate improved by less than 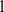
%. To evaluate the performance of the resulting feature set we used the Gini index 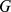, calculated with ROCR [29]. 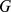 is related to the area under the curve of the true positive versus true negative rate (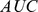) via 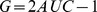, falling into the range 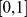. A value of 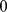 indicates random guessing and a higher value indicates a better separation power than random. Despite training on a specific day, the calculation of the Gini index 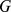 included the image feature data on all days, as we sought a FDA classifier which performs optimally on any given day. Per day and fold we thus obtained a Gini index 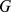 and we chose the feature set with the largest Gini index 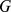.

Once the optimal feature set was determined, we selected, as before, each possible selection of nine out of 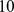 folds for training, and used the remaining fold on all days for evaluation of the Gini index. Hence, we obtained 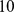 Gini indices per day, based on which we chose the day with the largest average 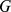. For all further verifications, we then trained five independent FDA classifiers with the obtained set of parameters by using two consecutive folds of the original 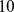 folds of the training data set. Thereby, each FDA was trained with only 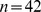 images per condition, which we expected to be a realistic sample size in biological applications. Moreover, this allowed assessment of the variation in the performance of the FDA classifier in the following, since all classifiers are statistically independent of one another. Finally, we determined a decision threshold 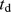 for each trained classifier. The strategy to determine the appropriate threshold depends on the actual application of the classifier. Here we chose to maximize the probability for a correct classification decision on all days and, hence, selected the decision threshold 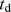 for which the sensitivity equals the specificity. Each of the five FDAs was trained on two folds and we used the data from the remaining eight folds of all days to set the decision threshold 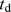, which we used for the downstream analysis.

## Results and Discussion

### Mixed Phenotype Cell Populations

**Figure 1:**
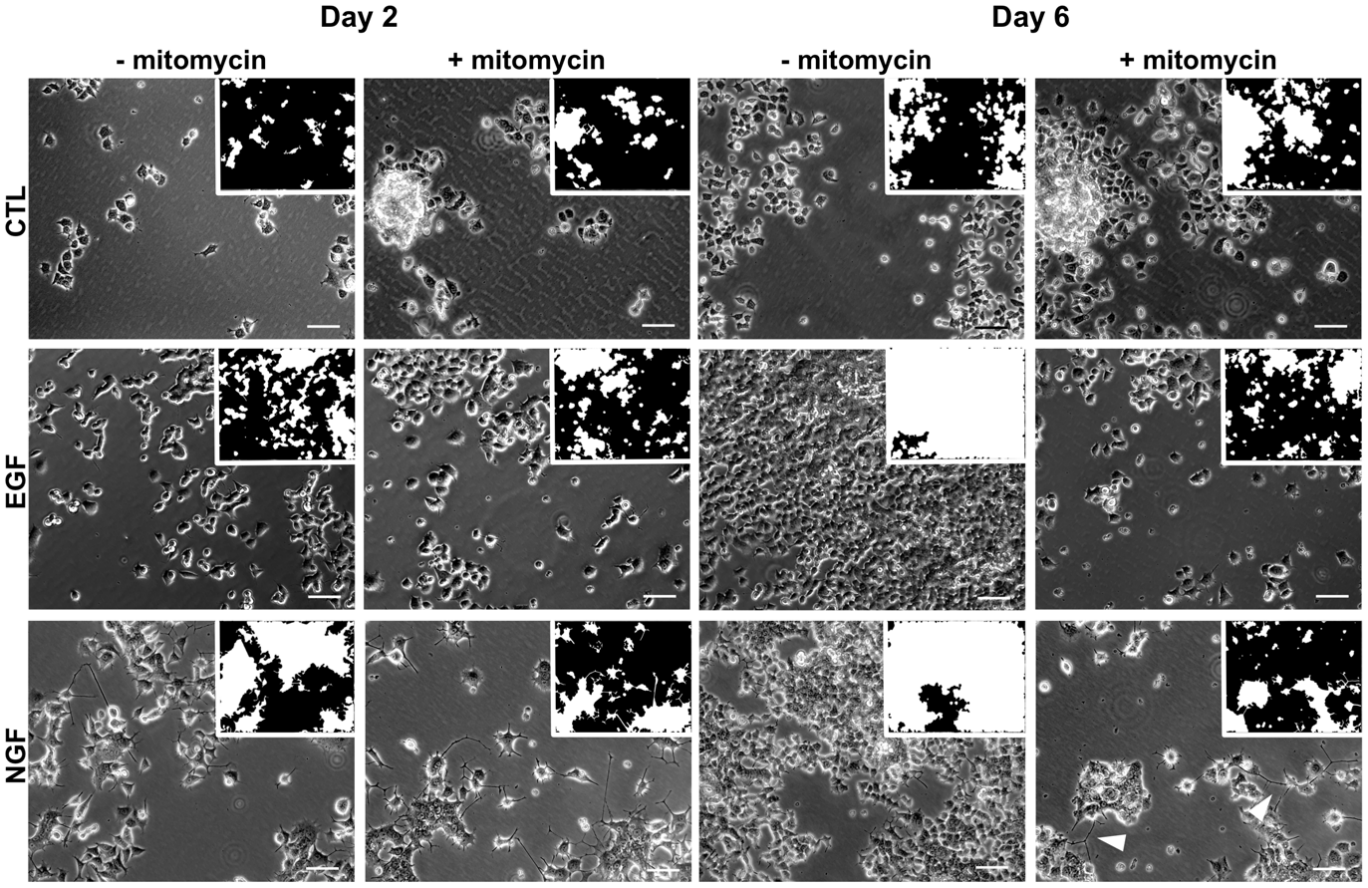
Phase contrast images of live-cells under the monitored conditions for days 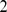 and 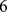 after treatment. The images have been selected using the FDA classifier that has its median FDA score on training day 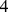. For clearer examples of the differentiated state, we selected for the NGF without mitomycin treatment images which correspond to the 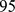
% quantile instead of the 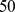
% median. Small inserts show the resulting ROI for each image. The white arrows indicate outgrown neurites on day 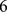. The proliferative effect of both EGF and NGF treatment without mitomycin is evident from the increased ROI on day 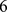. Mitomycin treatment inhibits proliferation and enhances the visibility of neurites. Bar, 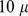
m.

PC12 cell populations treated with NGF respond with a mixed phenotype of both proliferation and differentiation as shown in Figure 1. Proliferation over six days was visible from the increased cell confluency (Figure 1, first and second rows). The hallmarks of differentiation are a flattened cell body followed by neurite outgrowth (Figure S1 and Figure 1). The latter, however, is much harder to detect, because these morphological changes are subtle on the population level, which shows over time an increased number of cells growing in clumps. To ensure that our image classifier distinguished the differentiation from the proliferation phenotype, we monitored PC12 cells under enhanced or reduced proliferation. PC12 cell proliferation is enhanced under EGF treatment [2], while mitomycin is known to suppress proliferation [30]. Under mitomycin treatment, apoptosis rates only increased slightly (Figure S2), while cell numbers were reduced over six days, irrespective of the stimuli (Figure 1,+mitomycin). As a side effect the numbers of cells growing in clumps were likewise reduced, thereby enhancing the neurite visibility of differentiating cells (Figure 1, Day 6, 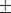 mitomycin).

**Figure 2:**
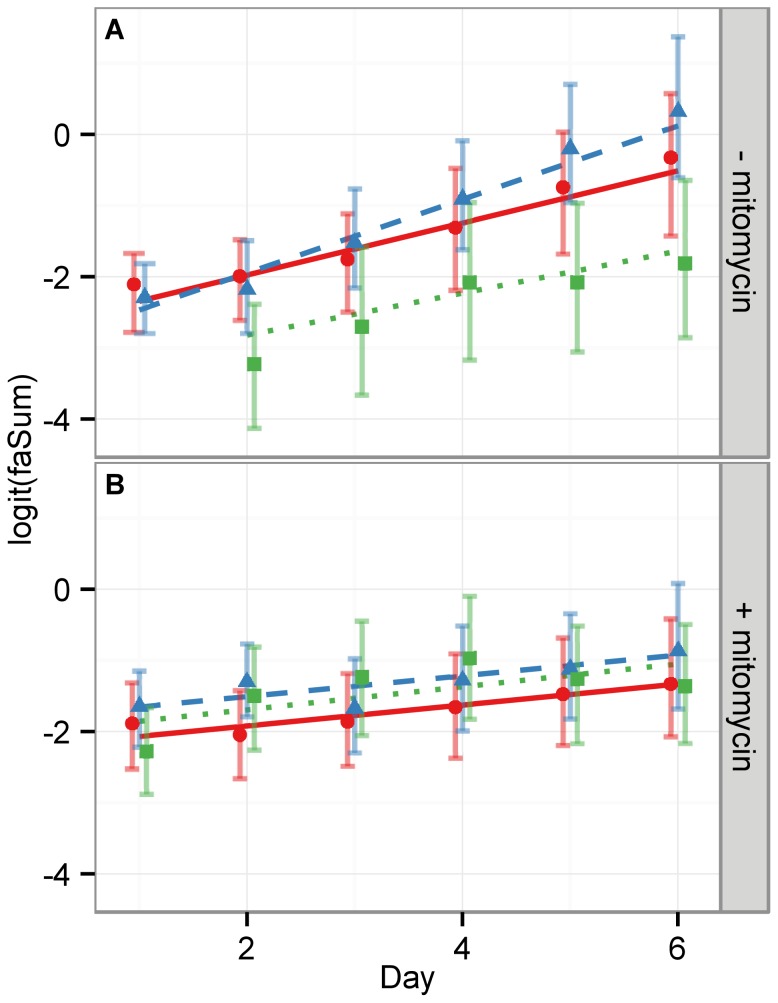
The image feature faSum measures qualitatively the cell area fraction occupying the well. As an approximation to cell content in a well, we show here the faSum feature. It corresponds to the white ROI area as shown in Figure 1. Since cells can vary in size this is only a qualitative measure of cell numbers. The upper panel (A) shows each condition without mitomycin treatment, whereas the lower panel (B) depicts the respective well with mitomycin treatment. Red circles and solid lines indicate the medians of the measurements of CTL cells, green squares and dotted lines of NGF stimulated cells and blue triangles and dashed lines of EGF stimulated cells. The error bars mark the 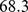
% c.i. and the lines depict linear regression analyses with slopes listed in Table 3. While the slopes of the regression lines in (B) with mitomycin are all statistically equivalent, the slope of the EGF treated cells in (A) is significantly larger then the respective NGF and CTL cells. This well reflects the biological known circumstance that EGF promotes proliferation.

To quantify the above observations we assumed that cell proliferation is proportional to cell confluency and hence used the image feature faSum (cf. Table. 2) to measure the image area covered by cells. Figure 2 shows a linear regression of the logit function for the faSum image feature under each condition (CTL, NGF and EGF, 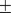 mitomycin). In the absence of mitomycin, the slopes of the regression lines for CTL and NGF stimulus are statistically equivalent, while the EGF stimulus displays the largest slope, i.e. the fastest proliferation, as depicted in Figure 1 (cf. Table 3). This supports the validity of the faSum image feature as a measure for proliferation. Under mitomycin treatment all slopes are significantly lower and of the same magnitude, indicating a strong decrease of proliferation in all experimental conditions with respect to no mitomycin treatment, as expected. Nevertheless, all slopes are still strictly positive, which indicates that the applied mitomycin concentration retained cell viability. This is in line with the observation from the micrographs (Figure 1).

**Table 3:** Proliferation kinetics.

	− mitomycin		+ mitomycin	
stimulus	slope [1/d]	σ [1/d]	slope [1/d]	σ [1/d]
CTL	0.382	0.012	0.151	0.012
NGF	0.358	0.016	0.165	0.012
EGF	0.587	0.012	0.154	0.012

Slopes of the linear regression lines for proliferation, see Figure 2.

### Detecting Neuronal Cell Differentiation

The extent of cell differentiation was easier to observe under mitomycin treatment. For this reason we evaluated the FDA on both data sets separately. However, the training of the FDA was carried out using only CTL and NGF stimulated cells without mitomycin treatment. Applying the training algorithm (cf. Material and Methods) we selected day 4 as the optimal training day and chose three image features. The selected features were the means of ecc, solidity and ext (cf. Table 2). Since all these features were related to the presence of large, partially filled regions, we suggest that the discrimination of the differentiated and the undifferentiated morphology occurs on the basis of how cell growth areas are physically connected. Undifferentiated cells grew in dense, yet disconnected colonies. In contrast, differentiating cells tend to connect these areas of high cell density by means of their extended neurites.

**Figure 3:**
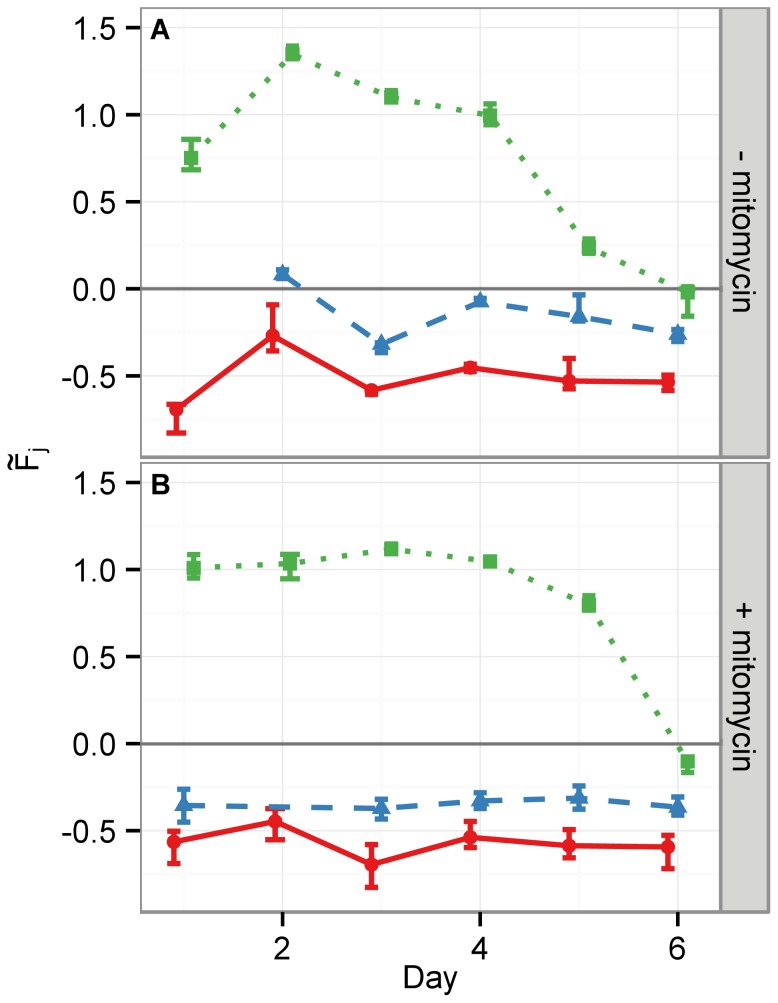
Median responses of the five independent FDA classifiers using the hold-out data set. The error bars depict the 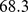
% c.i. Panels (A) and (B) show the FDA classifier response (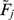) to cells without or with mitomycin treatment, respectively. The lines are shown to guide the eye and depict the measurement medians for the different treatment conditions. CTL: red circles and solid lines, NGF: green squares and dotted lines, EGF: blue triangles and dashed lines. The horizontal line through the origin 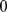 marks the decision threshold. FDA scores larger than 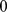 correspond to the differentiated cell status while scores below correspond to undifferentiated cells. Only on day 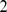 EGF treated without mitomycin in panel (A) are slightly above 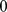, such that approximately 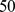
% of the cells under this condition are falsely classified as differentiated. However, NGF treated cells are always far away from the decision threshold 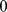 within the first four days. Hence, a more conservative threshold would remedy the false decision for EGF on day 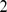 while still correctly classifying NGF treated cells.

In order to assess how variable independently trained FDA classifiers behave, we trained five independent classifiers based on the 10-fold split of the image data (cf. Material and Methods). Since each of these five FDA classifiers has its individual decision threshold 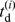, we standardized the scores 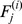 of each classifier 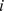 for image 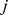 by
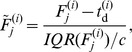
with 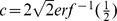 and the dispersion 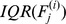 determined on the remaining 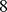-folds of the training data not used for training of the 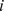
th FDA. Figure 3 summarizes the standardized responses of the five FDA classifiers on the hold-out data set by their medians. The FDA shows a high confidence (large distance from 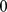) for classifying NGF treated cells within the first four or five days for cells treated without or with mitomycin, respectively. Due to the increasing cell confluency the detection of neurite structures became increasingly difficult at later days, decreasing the confidence of the FDA classifier. As this correlates with cell proliferation speed, image classification for differentiation worked longer in the case of mitomycin treatment. The unstimulated PC12 cells (CTL) were classified as non-differentiating on all days and under all treatments. The EGF condition was close to the decision threshold in absence of mitomycin. It was furthermore systematically closer to 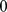 than CTL in the case of mitomycin stimulation, ultimately confirming that the early day FDA classifiers were picking up only the differentiation phenotype.

### Differentiation Detection Performance Assessment

**Figure 4:**
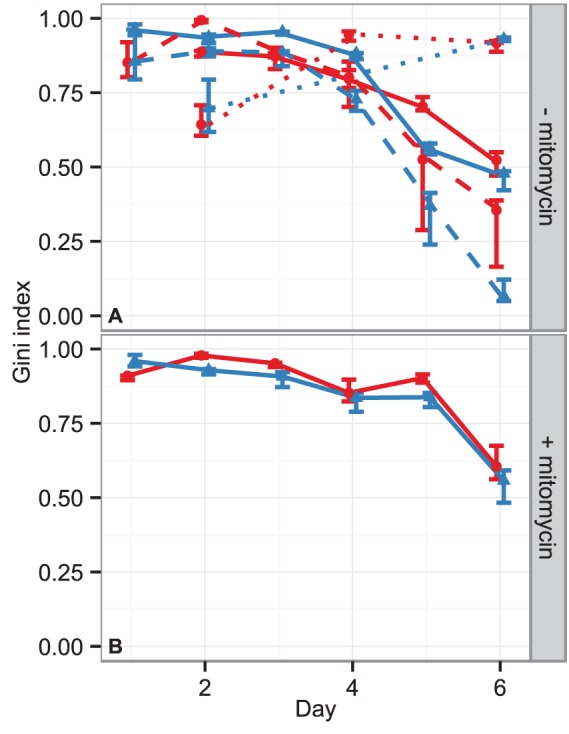
Comparison of Gini indices for the hold-out data, the technically independent data set, the biologically independent, and the experimentally distinct data set. (A) stimulation without mitomycin (B) stimulation under mitomycin treatment. The hold-out data and technically independent data sets are marked by the solid lines with blue triangles or red circles, respectively. Additionally, the dashed/dotted lines in panel (A) depict the biologically independent/experimentally distinct test sample, wherein the triangles and circles mark the respective technically independent replicates within this setup. Each point represents the median Gini index of the five independent FDA classifiers and the error bars mark the 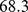
% c.i. A Gini index of 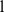 corresponds to perfect separation of differentiated and undifferentiated images. Since in the training data set and in the biologically independent data set the final cell density was high, the detection performance degrades such that the Gini index declines at later days. This is due to increasing build up of cell clumps rendering the differentiated cell morphology much harder to detect, even to the human eye. The experimentally distinct data set started from a lower cell density such that fewer cell clumps occurred, making the detection of differentiation feasible until day 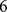.

We next verified the classifier performance on technically and biologically independent, as well as on experimentally distinct samples. As a first validation, we found the technically independent EGF stimulation (Figure 3) as being classified similar to CTL, because both showed a proliferation phenotype. We further classified a technically and a biologically independent replicate using the five independent FDA classifiers from above. The former replicate was measured in parallel with the training data in a separate cell culture well, the latter was measured in a different experimental run with a different cell passage. Moreover, we used an experimentally distinct sample, which was carried out with a much lower cell density of only 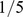 of the other experiments to mimic conditions of primary neuron culture, wherein cells grow individually, well separated from each other. Figure 4 depicts the Gini index of separation for all cases. The index stays above 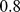 for all cases within the first four days and declines thereafter for the samples with high initial cell density. With mitomycin treatment, the Gini index is even larger than 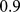 for the first 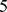 days. In addition, the experimentally distinct sample with a lower cell density shows no decrease for the Gini index after 4 days. This supports the conclusion that the FDA indeed learned the differentiation phenotype from the training data, observing the same effect as before that mitomycin pronounces the differentiated phenotype and suppressed proliferation.

**Figure 5:**
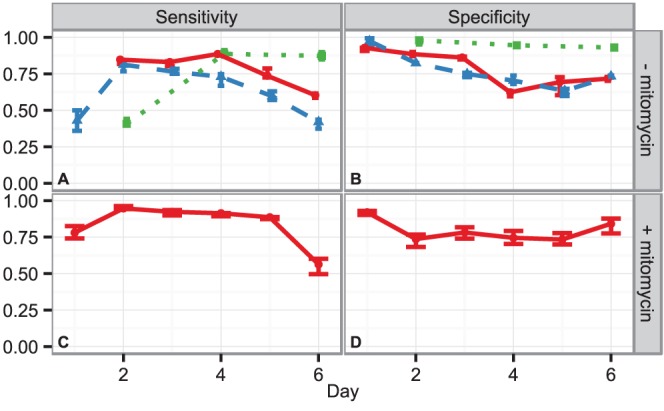
Sensitivity and specificity of the classifier, when considering NGF stimulated cells versus non stimulated CTL cells and EGF stimulated cells as negative reference. Sensitivity (A) and Specificity (B) for stimulation without mitomycin; (C) and (D) for stimulation under mitomycin treatment, respectively. The solid lines in panel (A) and (B) correspond to the technical replicate in the training data set. The dashed lines denote the biologically independent data set and include two technical replicates per condition. The dotted green line corresponds to the experimentally distinct data set at a lower cell density. Panels (C) and (D) show the results for the mitomycin treated cells from two technical replicates stemming from two cell culture wells. The symbols represent the median of the five independent FDA classifiers and error bars mark the 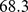
% c.i. While the overall performance for day 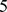 and 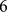 degrades in the case of no mitomycin for the experiments with higher cell density, the experimentally distinct data set conducted at 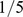 of the respective cell density does not show a degradation in detection performance as cell clumps are not growing and hence do not limit the detection of the differentiated phenotype.

To complement our verification we performed a sensitivity and specificity analysis based on the three independent data sets. We used in each case NGF treated cells as positive reference and used the CTL together with the EGF stimulated cells as negative reference. For all cases, the sensitivity and specificity of the FDA is in the range of 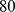
% until day 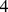 (see Figure 5A). Thereafter, the sensitivity declines without mitomycin treatment for the wells which were initially loaded with the higher cell density. Thereby, this asserts that the loss of detection performance is caused by cells growing in overlapping clumps, leading to a much more difficult detection of the differentiation phenotype. This is also shown with the third experimentally distinct sample. Due to the low cell number there is no decline of either sensitivity and specificity. The sensitivity on the other hand remains above 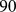
% in the case of mitomycin treatment until day 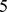 (see Figure 5C). The reason for the systematic shift of the specificity close to 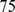
% is most likely due to the use of EGF as a further negative control. The FDA classifier systematically assigned scores to EGF stimulated cells that were closer to the decision threshold 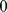, which led to a certain misclassification of EGF stimulated cells and a decrease in specificity.

### Sample Size Requirements

An important aspect for the applicability of our classification approach concerns sample sizes, i.e. the number of images required to be recorded for a statistical significant decision of cell differentiation status in a cell population. To this point we showed that classification results remained robust, i.e. the classifier response varied only marginally, using the training sample size of 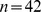 per condition. A FDA classifier is essentially the weighted sum of image feature values. Thus, we considered its result as a normally distributed variable. The sample size , i.e. the number of images per well, required to measure a mean response 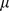, which is different from the decision boundary 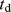 at a significance level of 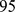
%, is at a sensitivity of 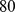
%
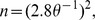
where 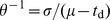 is the effect size. For a conservative estimate of 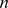, we considered the minimal effect size for days 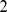 to 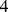 on the technically independent sample. We excluded days 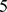 and 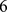, as the classification performance degraded due to the large cell confluency in the wells. The minimal effect size for cells stimulated with NGF in the absence of mitomycin was 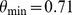 (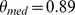, 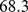
% c.i. 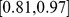) and was observed on day 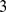. This leads to a minimal number of images of 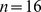 for the given experimental setup. However, of biological interest are usually conditions and treatments that alter the effect size, and thus, require a different sample size to reach statistical significance. Therefore, it is important to consider the scaling of 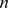 with the effect size. A rescaling of the effect size by a factor of 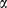 leads to an adjustment of 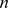 by 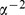. For example, treatment with mitomycin aids in the detection of differentiation, which is reflected by the larger resulting minimal effect size of 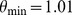 (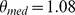, 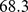
% c.i. 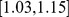) on day 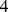. Therefore, only 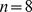 images are necessary under these conditions for a statistically significant test result at a sensitivity of 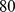
%. However, in the case of combinatorial cell treatments that decrease the effect size, e.g. if inhibiting downstream pathways of NGF, more images are required for the respective experimental setup. Hypothetically, if the effect size is halved, the resulting sample size must be 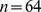, i.e. four times larger than without the inhibitor.

### Correspondence of Classifier Scores and Biological Differentiation Criterion

**Figure 6:**
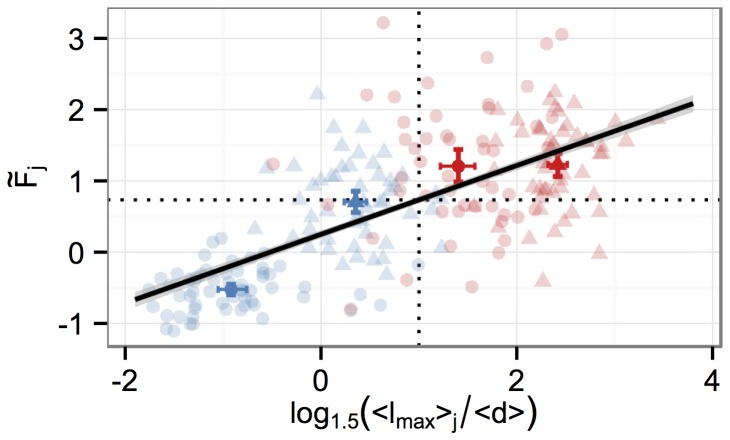
Correspondence of classifier score and biological differentiation criterion. Per cell we measured the longest neurites 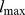 and diameters 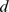 on day 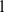 (blue) and 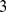 (red) without (circles) and with mitomycin (triangles) of cells receiving NGF stimulation. Transparent points represent individual cell and neurite length measurements per image and bold symbols depict the respective mean values (with 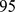
% c.i.) of the classifier score (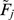) of image 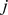 and the quantity 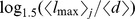 on the given day. If this value is larger than 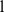, marked by the vertical dotted line, the length of the longest neurite of a cell is at least 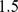 times the cell diameter. This is a commonly used biological threshold criterion for differentiation. The correlation with the respective FDA scores is 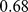 and the fitted linear regression line (black line, 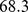
% c.i. shown in grey) has a positive slope of 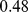. Therefore, the neurite length to cell diameter ratio is proportional to the classifier score. The threshold of 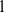 of the biological criterion corresponds to a threshold of 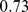 of the classifier on average (horizontal dotted line), i.e. choosing this threshold for the FDA will result in a decision of the FDA which is (on average) equivalent to the biological definition.

The biological definition if a cell is neuronally differentiated is traditionally based on the lengths of the cell’s neurites, which are optionally set in relation to the cell diameter. In the following, we define the quantitative measure of differentiation per cell as the ratio of the length of its longest neurite 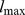 to the cell diameter 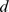. Whenever this ratio is larger than 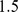 the cell is considered differentiated. Alternatively, 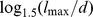 can be used, as a value larger than 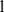 corresponds to differentiated and a value smaller than 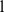 to undifferentiated cells. To show that our standardized FDA 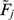 score is equivalent to this biological neuronal differentiation criterion, we manually measured cell neurites and diameters on the technical replicate well on day 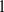 and 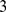 with and without mitomycin treatment. Due to locally high cell densities and difficulties in accurately measuring a cell diameter 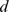, we determined a mean cell diameter of 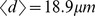 ([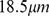
–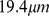] 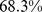 c.i.). Per image 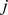 we measured the length of the longest neurite of representative cells. Since the classifier score 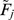 is determined per image (and not per cell within an image), we considered the mean value 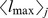 of all measured cells for an image 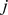. The Pearson correlation between 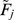 and 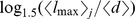 is 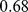 ([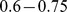] 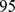
% c.i.), such that a linear association is a reasonable assumption. In Figure 6 the base data in the form 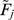 versus 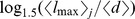 are shown together with their respective mean values of the four measured conditions and the linear regression line fitted to the base data. The 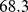
% c.i. of the linear line fit is shown in grey. The estimated slope is 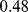 ([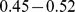] 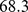
% c.i.) and thus differs highly significant from 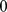. Therefore, a large mean neurite length 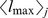 for cells within an image translates to a large score of the FDA. The threshold for the biological criterion is 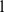 and can be converted with the linear fit into an associated classifier threshold, which is 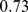 ([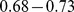] 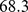
% c.i.). This value is somewhat higher than the standardized threshold of 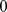. Hence, the biological criterion is conservative, requiring a clearer morphology than the classifier threshold of 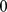. However, reconsidering the response of the FDA in Figure 3, one can observe that the FDA score is well above 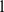 for the days 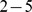 and hence is also compatible with the more conservative 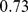 threshold.

### Conclusions

We have demonstrated the feasibility of detecting neuronal cell differentiation status in a live-cell population of mixed phenotype over a time-span of multiple days on the basis of phase contrast images. We accounted for the commonly observed heterogeneity in the image intensity, stemming from spatial scanning of the cell culture well, as well as from (re-)adjustment of instrument parameters during the experimental time window of one week. To compensate for these technical artifacts, we calibrated the ROI detection using empty cell culture wells and identified image features that are robust under intensity fluctuations. Apart from the technical challenges, classification of neuronal PC12 cell differentiation is furthermore hampered by the mixed phenotype response of simultaneous proliferation and differentiation. When stimulating PC12 cells with nerve growth factor (NGF), the cell population undergoes differentiation and proliferation. While we have shown that our classification approach detected differentiation in such phenotypically mixed populations, we considered a further treatment with mitomycin, thereby decreasing proliferation and consequently enhancing the detection of neuronal differentiation. This was reflected in our findings, as mitomycin increased the classification performance and improved the overall detection robustness. The detection performance measured in terms of the Gini index 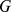 is larger than 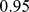 on the hold-out data set and 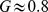 when considering an independent sample.

In summary, we have developed a novel data-driven, high-throughput, approach for monitoring cell differentiation status in label-free cell populations of a mixed phenotype. Importantly, we also showed that the classifier score is equivalent to the biological definition of cell differentiation if choosing an appropriate decision threshold such that the neurite length to cell body diameter must be greater than 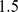. Furthermore, we showed that our classifier approach can detect cell differentiation also independently from the cell density of a cell population. We expect that the ability to monitor continuously live-cell populations for differentiation over days will prove valuable for large-scale analyses, e.g. in toxicity screens using neurite outgrowth measurement [31], [32] or Systems Biology analyses, when investigating dose-response behavior of single versus population-averaged cell behavior [33].

Building on one of the main achievements of this work to identify and verify suitable image features for classification of differentiation future steps will be the implementation of a regression to obtain a comprehensive description of the differentiation kinetics. This will enable *in vitro* studies to quantify the effect of inhibitors/stimulants on the rate of differentiation in label-free settings.

### Availability

All scripts for image analysis and statistical calculations are available to reviewers at http://www.zbsa.de/projekte/frias-hauke-busch/research/Neuron_Classification;

For application of the procedure in another study, the user should optimally supply a calibration and a training data set. These need to be registered within the software framework or simply replace the example data provided. With the calibrated and trained system successive experiments can readily be processed. The design of a user-friendly plugin to current Open Source microscopy software suites is currently in preparation and will be published in the near future.

## Supporting Information

Figure S1
**Immunostaining images of PC12 cells under NGF treatment and control conditions at days 1, 3, 5 after initial stimulation.** PC12 cells were labeled with a monoclonal Tubulin-Alexa488 mouse antibody (green). Cell nuclei were recognized by DAPI (blue). Cells were seeded at 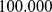 cells per 35 mm dishes and were treated with 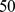 ng/ml NGF as described in Materials and Methods. Clearly, under NGF treatment, the outgrowth of neurites and a flatting of the cell body is visible. Bar, 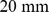.(TIF)

Figure S2
**Effect of mitomycin treatment on PC12 cells.** PC12 cells were treated with 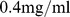 mitomycin or 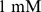 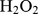 as positive control for cell death up to 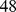 h. Samples were taken after 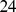 h and 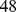 h, stained according to the Nicoletti method and subjected to specific DNA fragmentation by flow cytometry measurement. Data are representative of triplicates of three independent experiments (Error bars represent standard deviations). The stars denote a p-value 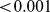 (one-sided t-test).(PDF)
