# Supplementary figures and images for: Label-Free Detection of Neuronal Differentiation in Cell Populations Using High-Throughput Live-Cell Imaging of PC12 Cells
Source: PLoS One. 2013 Feb 22;8(2):e56690. doi: 10.1371/journal.pone.0056690 (PMC3579923; doi:10.1371/journal.pone.0056690)

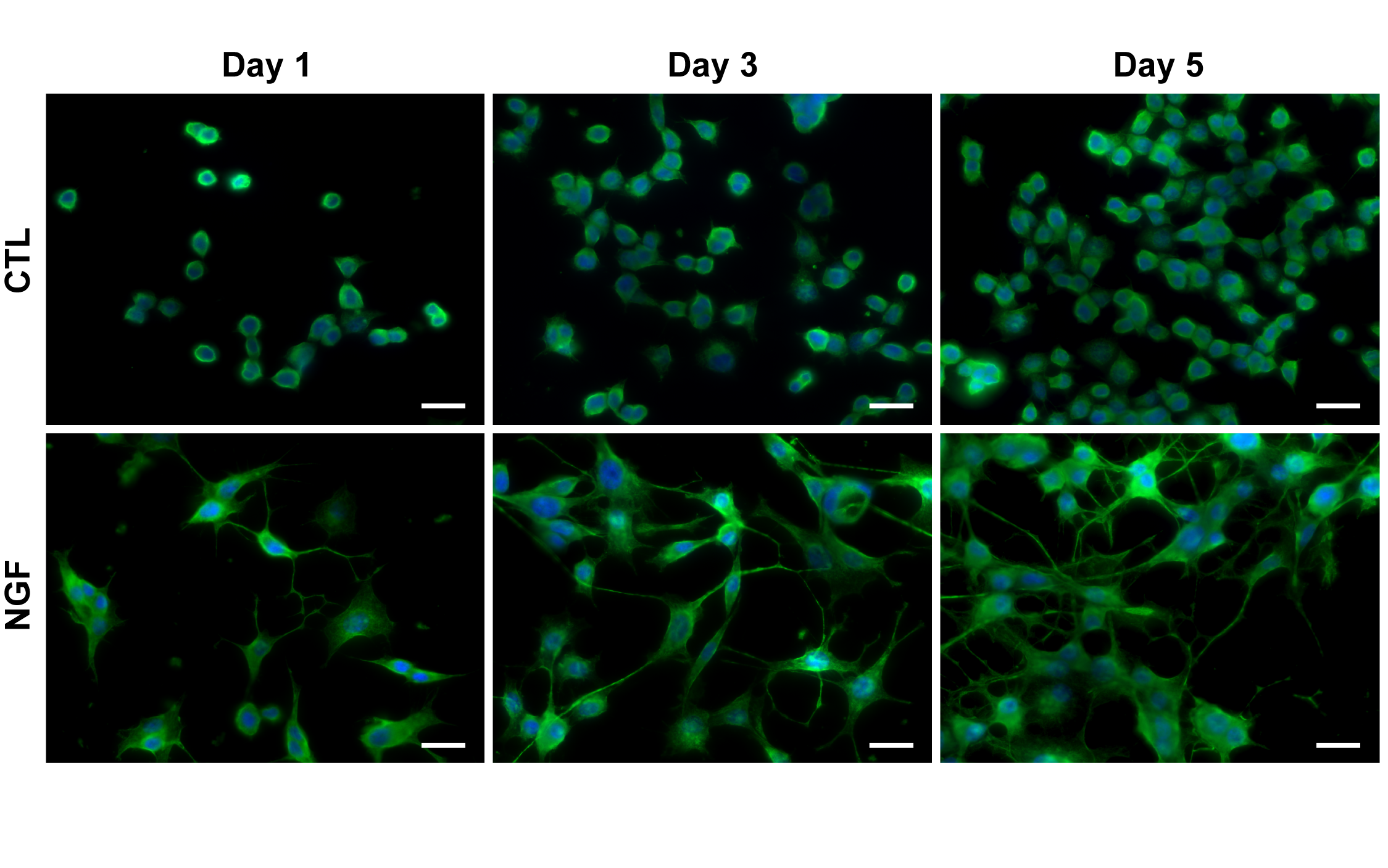

Supplement: Figure S1 — Immunostaining images of PC12 cells under NGF treatment and control conditions at days 1, 3, 5 after initial stimulation. PC12 cells were labeled with a monoclonal Tubulin-Alexa488 mouse antibody (green). Cell nuclei were recognized by DAPI (blue). Cells were seeded at cells per 35 mm dishes and were treated with ng/ml NGF as described in Materials and Methods. Clearly, under NGF treatment, the outgrowth of neurites and a flatting of the cell body is visible. Bar, . (TIF) [file pone.0056690.s001.tif]

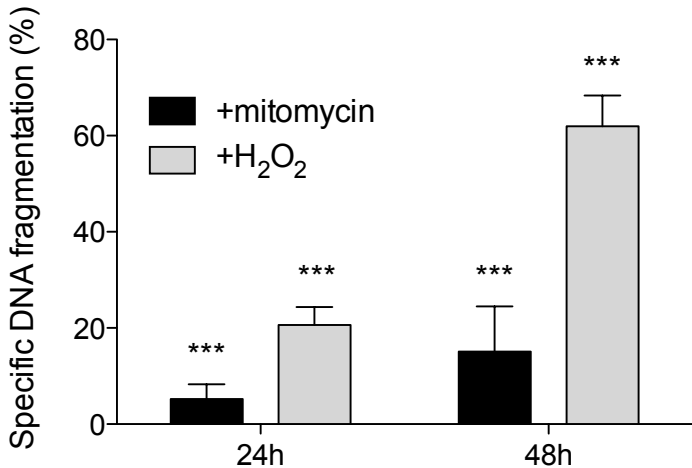

Supplement: Figure S2 — Effect of mitomycin treatment on PC12 cells. PC12 cells were treated with mitomycin or as positive control for cell death up to h. Samples were taken after h and h, stained according to the Nicoletti method and subjected to specific DNA fragmentation by flow cytometry measurement. Data are representative of triplicates of three independent experiments (Error bars represent standard deviations). The stars denote a p-value (one-sided t-test). (PDF) [file pone.0056690.s002.pdf]
